# Case Report: Successful Treatment With Monoclonal Antibodies in One APDS Patient With Prolonged SARS-CoV-2 Infection Not Responsive to Previous Lines of Treatment

**DOI:** 10.3389/fimmu.2022.891274

**Published:** 2022-06-21

**Authors:** Beatrice Rivalta, Donato Amodio, Carmela Giancotta, Veronica Santilli, Lucia Pacillo, Paola Zangari, Nicola Cotugno, Emma Concetta Manno, Andrea Finocchi, Stefania Bernardi, Luna Colagrossi, Leonarda Gentile, Cristina Russo, Carlo Federico Perno, Paolo Rossi, Caterina Cancrini, Paolo Palma

**Affiliations:** ^1^ Research Unit of Primary Immunodeficiencies, Immune and Infectious Diseases Division, Academic Department of Pediatrics (DPUO), Bambino Gesù Children’s Hospital, Istituto di Ricovero e Cura a Carattere Scientifico (IRCCS), Rome, Italy; ^2^ Department of Systems Medicine, University of Rome “Tor Vergata”, Rome, Italy; ^3^ Research Unit of Clinical Immunology and Vaccinology, Academic Department of Pediatrics (DPUO), Bambino Gesù Children’s Hospital, Istituto di Ricovero e Cura a Carattere Scientifico (IRCCS), Rome, Italy; ^4^ Microbiology and Diagnostic Immunology Unit, Bambino Gesù Children’s Hospital, Istituto di Ricovero e Cura a Carattere Scientifico (IRCCS), Rome, Italy; ^5^ Multimodal Medicine Research Area, Bambino Gesù Children’s Hospital, Istituto di Ricovero e Cura a Carattere Scientifico (IRCCS), Rome, Italy

**Keywords:** SARS-CoV-2, COVID19, IEI, activated PI3K delta syndrome (APDS), monoclonal antibody, remdesivir, long-lasting infection, APDS

## Abstract

We described the case of a patient affected by activated PI3K-kinase delta syndrome (APDS) and a long-lasting and pauci-symptomatic SARS-CoV-2 infection, treated with multiple therapeutic agents including remdesivir and SARS-CoV-2-neutralizing monoclonal antibodies. We detected the clearance of the virus 105 days from the first positive swab and 7 days after monoclonal antibody administration. At genotyping, the SARS-CoV-2 virus resulted as wild type on all samples tested. This case shows the monoclonal antibodies’ good tolerability and efficacy in reducing viral shedding in long-lasting infections refractory to other treatments.

## Introduction

The outcome of COVID-19 in patients with an inborn error of immunity (IEI) is influenced by the underlying disorder and comorbidities. Complications including respiratory failure, cardiac involvement, and thromboembolism are mainly related to a dysregulated immune response (1–9). Long-lasting infections are associated with the emergence of SARS-CoV-2 variants overall in immunosuppressed patients (10). We describe the case of a 25-year-old man affected by activated PI3K-kinase delta syndrome (APDS) with a long-lasting and pauci-symptomatic SARS-CoV-2 infection initially treated with remdesivir who eventually achieved viral clearance after treatment with SARS-CoV-2-neutralizing monoclonal antibodies.

## Case Description: Diagnostic Assessment and Therapeutic Intervention

**Figure 1:**
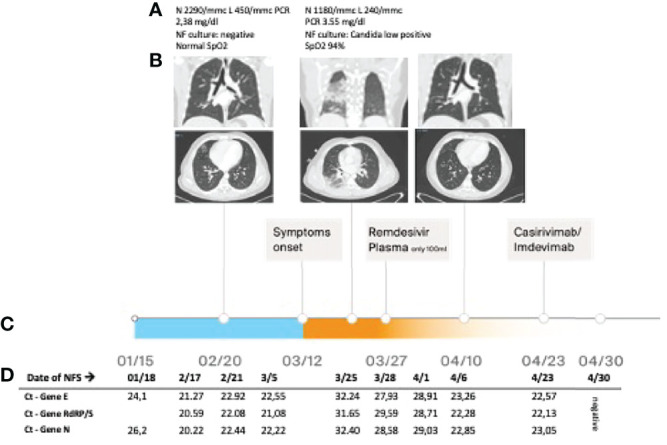
**(A)** Blood exams, nasopharyngeal (NF) culture result, and SpO_2_ during the episodes; **(B)** lung CT scan performed at different time points; **(C)** timeline; and **(D)** real-time PCR cycle threshold (Ct) of SARS-CoV-2 on nasal swab.

The patient was diagnosed with APDS at 16 years of age (mutation E1021K in *PI3KCD*). Since childhood, he presented recurrent otitis and sino-pulmonary infections, a chronic EBV infection, lymphadenopathy, an episode of hemolytic anemia, and three episodes of pericarditis. Since his first years of life, he was treated with endovenous immunoglobulin (Igev) replacement therapy, respiratory therapy, cycles of antibiotics, steroids, and rituximab. In 2016, he was treated for a diffuse large B lymphoma (DLBCL) with rituximab, cyclophosphamide, doxorubicin, vincristine, and prednisone (R-CHOP) with complete remission. In June 2020, he was diagnosed with a relapsed DLBCL and a pulmonary atypical mycobacterial infection, confirmed by molecular assay (PCR and probe hybridization) on bronchoalveolar lavage fluid specimen. He was treated with rituximab, ibrutinib, and bendamustine until December 2020 and a triple-drug anti-mycobacterial regimen (azithromycin, rifampicin, and ethambutol) for 1 year until June 2021 (11). A few days after contact with a positive family member on January 15, 2021, an antigenic test on a nasal swab (NS), performed as a screening for SARS-CoV-2 required to perform routine investigations, resulted positive. The positivity was confirmed on January 18, 2021, by RT-PCR on an NS (**Figure 1**). A thorax CT scan on February 20 revealed a sub-pleural pulmonary consolidation, in the absence of symptoms. The RT-PCR on the NS resulted still positive for SARS-CoV-2, and the cultures on nasopharyngeal aspirates and blood resulted negative. He was treated with piperacillin–tazobactam and Igev. Subsequent RT-PCR performed on NS documented the asymptomatic infection persistence (**Figure 1**) until mid-March when he presented low-grade fever, dry cough, chest pain, and exertional dyspnea associated with a worsening of pulmonary consolidation. The culture on nasopharyngeal aspirates showed a low positivity for candida, and blood culture resulted negative. Due to the persistence of SARS-CoV-2 infection documented on SARS-CoV-2 RNA detection on NS and a low-positive serology (low positivity for anti-S and negative for anti-N), at the end of March he was treated with remdesivir for 10 days with good tolerability except for a mild increase in transaminases. Unfortunately, it had a low impact on viral replication as documented by low cycle threshold (Ct) values in RT-PCR performed on the NS (**Figure 1**) and viral load quantification ranging from 10^3^ to 10^5^ cp/ml (ORF8 and RdRp genes). In the attempt of reaching viral clearance, the patients received a COVID‐19‐vaccinated plasma infusion obtained by a single donor recently vaccinated for SARS-CoV-2 and a level of anti-S abs of 916 U/ml determined by electrochemiluminescence (12). Unfortunately, the patient developed an adverse event characterized by fever, diffuse skin rash, and dyspnea and the infusion was interrupted after a few minutes. The CT scan performed on April 10 showed almost complete remission of pulmonary consolidation. Considering the persistence of high viral load in NS (**Figure 1**) on April 23, he was treated with SARS-CoV-2-neutralizing monoclonal antibodies REGEN-COV (casirivimab and imdevimab) with good tolerability and efficacy. The SARS-CoV-2 test on NS performed on April 30 and May 12 turned negative (**Figure 1**). According to epidemiology tracking, SARS-CoV-2 strain circulation at the time was mostly represented by the wild-type lineage, the Alpha (B.1.1.7 VOC-202012/01 genomes), Beta, and Delta variants of concern. By using SNP genotyping, no S mutation of SARS-CoV-2 (E484K, E484Q, 501Y, K417N, L452R) was detected on all samples tested. Characterization analysis was concluded as wild-type virus.

During the observation, mild neutropenia (nadir 1,020/mmc) and the worsening of the preexisting lymphopenia were documented (nadir 240/mmc). The immunophenotype showed a marked reduction of CD4+ T cells (8.9% of lymphocytes) and the absence of CD19+ B cells, as expected after the recent therapy with rituximab. A mild reduction of IgA (34 mg/dl) with normal IgM (72 mg/dl) and IgG (637 mg/dl before infusion) was also documented (**Supplementary Material Table 1**).

After 1 year from the SARS-CoV-2 infection, the patient was currently free from any symptom related to long-lasting COVID-19. The last serology showed a low level of anti-S and anti-N antibodies, potentially influenced by the immunoglobulin replace therapy. Since the patient refuses to undergo SARS-CoV-2 vaccination and considering the current treatment with brentuximab due to a newly diagnosed lymphoproliferative disease, we decided to administer tixagevimab+cilgavimab at the beginning of May 2022.

## Discussion

Patients with IEIs are prone to frequent and severe infections. In the last months, some authors have analyzed in large international cohort studies the course of SARS-CoV-2 infection in patients with IEIs (1–9) and their response to vaccination (13–15). Most patients with IEIs develop a mild disease but may present persistent viral replication, and younger individuals with IEIs are more frequently admitted to the ICU compared to the general population. Moreover, patients with combined immunodeficiency and IEIs with immune dysregulation show a worse outcome (1, 2, 6, 8).

A multicenter study conducted on previously healthy patients with severe COVID-19 has found a high incidence of innate errors of TLR3 and the IRF-7-dependent pathway, responsible for low serum type I IFNs (16). Moreover, Bastard et al. have found neutralizing autoantibodies against type I IFNs (especially INFω and INFα) (17), suggesting a major role of IFN I pathways in the disease course.

APDS is characterized by a hyperactivation of the PI3K-AKT-mTOR pathway. These patients present recurrent sinopulmonary and chronic herpes virus infections, in some cases persistent granulomatous skin lesions associated with BCG vaccination (18, 19) and immune dysregulation manifestations (cytopenia, arthritis, colitis, and lymphoproliferation) with a high risk for lymphoma (19, 20). In these patients, high expression and secretion of INFγ by CD8+ (21), CD4+ T, and Tfh T cells (22) and a high plasmatic level of INFγ (23) though with impaired cytotoxic activity against viruses like EBV have been reported.

Few data about the impact and treatment of SARS-CoV-2 infection in patients with IEIs are available. The expansion of prophylactic therapy for SARS-CoV-2 in patients with poor immune responses is increasingly prudent. Poor vaccine immunogenicity and the growing threat of mutational escape globally are forcing the development of alternative targets in the form of monoclonal antibodies. Some international case studies report mild symptomatology in APDS patients with COVID19. Meyts et al. reported the case of one APDS patient with a mild symptomatic SARS-CoV-2 infection who obtained viral clarence within 2 weeks. Other authors reported some other APDS patients with mild symptoms associated with SARS-CoV-2 infection, but no data on viral clarence timing were reported (1, 3, 24). In some patients with IEIs and severe SARS-CoV-2 infection, mainly with humoral IEIs, or patients with B cell deficiency previously treated with rituximab, treatments with convalescent plasma or monoclonal antibodies, in some cases in combination with remdesivir, resulted in a significant improvement of symptoms, although viral persistence may be variably affected (1, 25–27). Considering the high incidence of long-lasting infections in patients with IEIs, the efficacy and indication of monoclonal antibody or convalescent plasma therapies, which in the general population are limited to the first days of infection, could be extended to the later stage (1, 25–27). Moreover, long-lasting infection associated with the emergence of SARS-CoV-2 variants is reported in immunosuppressed patients (10) and antiviral or monoclonal/plasma therapies may act as selective pressures favoring the emergence of new variants (28, 29). In our patient, both the long-lasting infection and different administered therapies were not associated with the onset of new variants. Different factors such as the recent chemotherapy and the complex IEI phenotype could have contributed to the long-lasting infection and possibly to the mild symptomatology associated with the infection. SARS-CoV-2-neutralizing monoclonal antibodies have proven effective in obtaining viral clearance even if administered far from the beginning of the infection. The present case highlights the potential benefit of REGN-COV as therapy for persistent SARS-CoV-2 infection in APDS patients, although with the caveat that clinical efficacy against new variants needs to be explored *in vitro* and *in vivo* studies. Interestingly, Takashita et al. very recently showed in vitro the potential efficacy of REGEN-COV against the currently circulating Omicron BA.2 variant (30) More studies on larger cohorts of patients are needed to assess the efficacy of these therapies, their proper dosage and use in combination with other antiviral treatments, in SARS-CoV-2 infected patients with IEIs and particularly in IEIs with immunodysregulation.

## Data Availability Statement

The original contributions presented in the study are included in the article/**Supplementary Material**. Further inquiries can be directed to the corresponding author.

## Ethics Statement

The examinations were carried out in accordance with the Declaration of Helsinki. The patient gave written informed consent for the use of therapies and for the publication of pseudonymized reports.

## Author Contributions

BR and DA analyzed the clinical data and drafted the manuscript. CG, VS, LP, PZ, NC, and EM were involved in the patient’s clinical care. AF, SB, PR, CC, and PP were involved in the patient’s clinical care and supervised the work. LC, LG, CR, and CP performed and interpreted the virological data. All authors contributed to the article and approved the submitted version.

## Funding

This study was supported by “Fondo 5x1000” to DA.

## Conflict of Interest

The authors declare that the research was conducted in the absence of any commercial or financial relationships that could be construed as a potential conflict of interest.

## Publisher’s Note

All claims expressed in this article are solely those of the authors and do not necessarily represent those of their affiliated organizations, or those of the publisher, the editors and the reviewers. Any product that may be evaluated in this article, or claim that may be made by its manufacturer, is not guaranteed or endorsed by the publisher.
