# Supplementary material for: Case Report: Successful Treatment With Monoclonal Antibodies in One APDS Patient With Prolonged SARS-CoV-2 Infection Not Responsive to Previous Lines of Treatment
Source: Front Immunol. 2022 Jun 21;13:891274. doi: 10.3389/fimmu.2022.891274 (PMC9253383; doi:10.3389/fimmu.2022.891274)
Supplement: Supplementary file 1 [file DataSheet_1.docx]

# Supplementary material

|  | **24 y** | **25y**  **during infection** | **25y**  **4 months after the infection** | **25y**  **1 year from infection** |
| --- | --- | --- | --- | --- |
| Total Lymphocyte count 10^3^/uL | **1280**  (1400-4200) | **450**  (1400-4200) | **710**  (1400-4200) | **460**  (1400-4200) |
| CD3+ CD45+ | 79,6  (50-91) | 90  (50-91) | 82,1  (50-91) | 65,7  (50-91) |
| CD3− CD16+CD56+ | 9  (5-49) | 9,7  (5-49) | 7,1  (5-49) | 25,9  (5-49) |
| CD3+ CD4+ | **21,5**  (28-64) | **8,9**  (28-64) | **15,1**  (28-64) | **16,9**  (28-64) |
| CD3+ CD8+ | **57,2**  (12-40) | **80,5**  (12-40) | 65,3  (12-40) | **47,6**  (12-40) |
| CD19+ CD45+ | 9,2  (6,6-10,8) | 0  (6,6-10,8) | 9,7  (6,6-10,8) | 7,7  (6,6-10,8) |
| % CD3 lymphocyte subsets | | |  |  |
| TCR𝛼/𝛽+ | 98,8  (44-92) |  | 99,4  (44-92) |  |
| TCR𝛾/𝛿+ | **1,15**  (2-24) |  | 0,4  (2-24) |  |
| CD4− CD8− | 0,5  (0,57-5) |  | 0,4  (0,57-5) |  |
| % CD4 lymphocyte subsets | | |  |  |
| CD27+CD45RA+ Naïve | **4**  (16-100) |  |  | 41,4 |
| CD31+CD45RA+ (RecentThymicEmigrants, RTE) | **2**  (7-100) |  |  | 0,8 |
| CD27+CD45RA−  (CentralMemory) | 72,1  (18-95) | **98,9** | **92,1** | **98,9** |
| CD27−CD45RA−  (EffectorMemory) | 23,5  (1-23) |  |  |  |
| CD27−CD45RA+ (EffectorMemoryCD45RA+, EMRA) | 0,35  (0,0031-1,8) |  |  | 3,3 |
| CD25+CD127lowFOXP3+ (RegulatoryTcell, Treg) | 3,93  (4-17) |  |  |  |
| CD45RO+ CXCR5+  (Follicular helper T cell, Tfh) | 7,4  (5-56) |  |  |  |
| % CD8 lymphocyte subsets | | |  |  |
| CCR7+CD45RA+ (Naïve) | **2,6**  (6-100) |  |  |  |
| CCR7+CD45RA−  (Central Memory) | **0,5**  (1-20) | **66** | **71** | **74** |
| CCR7−CD45RA− (EffectorMemory) | 68,8  (14-98) |  |  |  |
| CCR7−CD45RA+ (EffectorMemoryCD45RA+, EMRA) | 28,1  (7-53) |  |  |  |
| % B lymphocyte subsets | | |  |  |
| CD24++CD38++ (Transitional) | **52**  (3-5,9) | **-** | **38.9**  (3-5,9) | **-** |
| CD27−IgD+IgM+ (Naïve) | 76,6  (65,6-79,6) | - | 29,8  (65,6-79,6) | - |
| CD27+IgD+IgM+  (Unswitched memory) | 10,6  (7,4-13,9) | - | 14  (7,4-13,9) | - |
| CD27+IgD−IgM−  (Switched memory) | **5,1**  (7,2-12,7) | **-** | **0**  (7,2-12,7) | **-** |
| CD27− IgD− IgM− | **7,7**  (2,1-4,4) | **-** | **-** | **-** |
| CD21low CD38low | 1,3  (0,9-3,1) | - | - | - |
| IgG (mg/dl) | 727 * | 647* | 695* | **466*** |
| IgA (mg/dL) | 48 | 34 | 23 | **12** |
| IgM (mg/dL) | **326** | 72 | **205** | 134 |

**Table 1:** Immunological characterization of the patient before chemotherapy and infection and during infection. * During Ig replace therapy. Range value in the bracket. [Morbach H, Eichhorn EM, Liese JG, Girschick HJ. Reference values for B cell subpopulations from infancy to adulthood. Clin Exp Immunol. 2010;162:271–9; Garcia-Prat M, Álvarez-Sierra D, Aguiló-Cucurull A, Salgado-Perandrés S, Briongos-Sebastian S, Franco- Jarava C, et al. Extended immunophenotyping reference values in a healthy pediatric population. Cytom Part B Clin Cytom [Internet]. 2019;96:223–33; Schatorjé EJH, Gemen EFA, Driessen GJA, Leuvenink J, van Hout RWNM, de Vries E. Paediatric Reference Values for the Peripheral T cell Compartment. Scand J Immunol [Internet]. 2012;75:436–44.]

| Date | Anti-N (ECLIA) | Anti-S (ECLIA) |
| --- | --- | --- |
| February 16th | Absent | Absent |
| March 25th | Absent | Absent |
| April 1st | Absent | 8,95U/mL |
| April 9th | Absent | 5,72 |
| April 14th | Absent | 4,75 |
| May 9th | Absent | 151 |
| May 26th | Absent | 233 |
| September 10th | Absent | 184 |
| April 6th | Present | 187.5 |
| May 3rd | Present | 89,3 |

**Table2:** SARS-Cov2 Antibodies during infection and after 4 months from infection. The low positivity of serology detected at the end of March may be related to the small volume of plasma from a single donor recently vaccinated for SARS-CoV-2 and immunoglobulin infusions.
